# Supplementary material for: Understanding asymmetric synergistic effect between movie actors
Source: PLoS One. 2023 Apr 20;18(4):e0284613. doi: 10.1371/journal.pone.0284613 (PMC10118101; doi:10.1371/journal.pone.0284613)
Supplement: Attachment — Submitted filename: Response.docx [file pone.0284613.s001.docx]

Response to Reviewers

We firstly thank all the reviewers for their useful comments. According to the comments, we have carefully revised the paper.

Reviewer #1: The manuscript is well-organized and clearly written, with a well-defined research question and hypothesis. The methods are rigorous and appropriate for addressing the research question, and the results are clearly presented and supported by the data. I particularly appreciate the thoroughness of the discussion section, which provides a comprehensive analysis of the findings and their implications.
Overall, I believe that the manuscript is a valuable contribution to the field, and I am confident that it will be well-received by the readership of PLOS ONE. I look forward to seeing it published.

Reply: Thank you very much for your appreciation.

Reviewer #2: The purpose of the study and the results of the experiments are well listed. It seems that the strengths and weaknesses of the thesis have been well considered.
It is considered that it should be submitted after final review.

Reply: Thank you very much for your positive comments.

Reviewer #3: - The manuscript sounds great and the conclusions are drawn properly based on the data.
- The research and study are performed suitably and rigorously.
- The data underlying the methods in their manuscript is fully available.
- The manuscript is presented in standard English.
- Regarding the form, please align the text.

Reply: Thank you very much for your positive comments.

We have carefully checked the text area in the paper, and align them.

Reviewer #4: The ideas and contributions of the research is presented quite clearly. However, I have some objective assessments to improve the quality of the article as follows:

Reply: Thank you very much for your positive comments.

1. The structure has been clearly divided into sections including an introduction, presentation of the proposed method, experimental results, evaluation, and conclusion.

Reply: Thank you very much for your positive comments.

However, related studies have not been introduced in detail and should be described in a separate section immediately after the Introduction.

Reply: Thank you for your comments. We have considered this comment very carefully. Different from the other journals, most papers in Plos One has no “Related Work” section. Thus, we have decided to put more related work in “Introduction” section, instead of making a new section for “Related Work”.

2. The parameters and variables in the equations should be described in detail which is easy to understand. Authors should write paragraphs containing these equations to increase the coherence of the paper. Besides, describing notations in a table is effective in reading comprehension of formulas.

Reply: Thank you for your comments. We have considered this comment very carefully. Since we have only small number of parameters (which can be easily understood), we have decided NOT to put additional description.

3. Descriptions for tables and figures should be placed next to this information in the form of captions so that readers can quickly grasp the information.

Reply: Thank you for your comments. We have considered this comment very carefully. Since this paper has been working with LaTex, the Figures & Tables are automatically placed. In addition, the editorial work will be supported once the paper is accepted.

4. Some passages are written from the author's subjective point of view and have not been mentioned by citations from other articles. Adding citations in the sentences is important to increase the credibility of the article.

Reply: Thank you for your comments. We have considered this comment very carefully. In order to improve the credibility of this paper, we have added the following references

- Assenova VA (2018) Modeling the diffusion of complex innovations as a process of opinion formation through social networks. PLoS ONE 13(5): e0196699.
- Pirasteh P et al. (2015) Exploiting Matrix Factorization to Asymmetric User Similarities in Recommendation Systems. Knowledge-Based Systems 83:51-57.
- Grabowicz PA, Ramasco JJ, Moro E, Pujol JM, Eguiluz VM (2012) Social Features of Online Networks: The Strength of Intermediary Ties in Online Social Media. PLoS ONE 7(1): e29358. https://doi.org/10.1371/journal.pone.0029358

5. The author should explain the overview of the major sections, identify the main points in these sections and should pay attention to writing a title that can better cover the content of that section. For example "Evaluation" and "Results and discussion" sections should be explained in detail, the title "Using data" is not really comprehensive, etc.

Reply: Thank you for your comments. We have considered this comment very carefully. The titles of those sections have been corrected.

The above comments are my reviews of this paper, I hope to contribute my efforts to complete high-value research.
